# Recurrent headache and interpersonal violence in adolescence: the roles of psychological distress, loneliness and family cohesion: the HUNT study

**DOI:** 10.1186/1129-2377-15-35

**Published:** 2014-06-10

**Authors:** Synne Øien Stensland, Siri Thoresen, Tore Wentzel-Larsen, John-Anker Zwart, Grete Dyb

**Affiliations:** 1 Norwegian Centre for Violence and Traumatic Stress Studies, University of Oslo, P.B. 181, Nydalen 0409 Oslo, Norway; 2 Centre for Child and Adolescent Mental Health, Eastern and Southern Norway, University of Oslo, Oslo, Norway; 3 Institute of Clinical Medicine, University of Oslo, Oslo, Norway; 4 Department of Neurology/FORMI, Oslo University Hospital, Oslo, Norway

**Keywords:** Interpersonal violence, Sexual abuse, Bullying, Loneliness, Social isolation, Psychological distress, Family cohesion, Social support, Recurrent headache

## Abstract

**Background:**

Recurrent headache is the most common and disabling pain condition in adolescence. Co-occurrence of psychosocial adversity is associated with increased risk of chronification and functional impairment. Exposure to interpersonal violence seems to constitute an important etiological factor. Thus, knowledge of the multiple pathways linking interpersonal violence to recurrent headache could help guide preventive and clinical interventions. In the present study we explored a hypothetical causal model where the link between exposure to interpersonal violence and recurrent headache is mediated in parallel through loneliness and psychological distress. Higher level of family cohesion and male sex is hypothesized to buffer the adverse effect of exposure to interpersonal violence on headache.

**Methods:**

The model was assessed using data from the cross-sectional, population-based Young-HUNT 3 study of Norwegian adolescents, conducted from 2006–2008. A cohort of 10 464 adolescents were invited. The response rate was 73% (7620), age ranged from 12 and 20 years, and 50% (3832) were girls. The study comprised self-report measures of exposure to interpersonal violence, loneliness, psychological distress and family cohesion, in addition to a validated interview on headache, meeting the International Classification of Headache Disorders criteria. Recurrent headache was defined as headache recurring at least monthly during the past year, and sub-classified into monthly and weekly headache, which served as separate outcomes.

**Results:**

In Conditional Process Analysis, loneliness and psychological distress consistently posed as parallel mediating mechanisms, indirectly linking exposure to interpersonal violence to recurrent headache. We found no substantial moderating effect of family cohesion or sex.

**Conclusions:**

Loneliness and psychological distress seem to play crucial roles in the relationship between exposure to interpersonal violence and recurrent headache. To facilitate coping and recovery, it may be helpful to account for these factors in preventive and clinical interventions. Trauma-informed, social relationship-based interventions may represent a major opportunity to alter trajectories of recurrent headache.

## Background

Recurrent headache disorders, such as migraine and tension-type headache, are the most common and disabling pain conditions in childhood and adolescence [1]. The prevalence rates are higher in females, especially after transition to puberty [2]. Lack of parental support and poor family function is associated with chronification of headache [3] and higher pain-related disability [4]. Further, co-occurrence with psychological problems is common, and increase risk of functional impairment and persistence of headache complaints into adulthood [5-10]. Recent epidemiological and clinical research indicate that exposure to interpersonal violence, such as violence, sexual abuse, and bullying, may contribute to onset, persistence or exacerbation of headache complaints, in interplay with genetic predispositions [11-18]. Knowledge of pathways linking interpersonal violence exposure to recurrent headache could help guide prevention and tailor intervention. However, the multiple pathways linking adverse social interaction such as exposure to interpersonal violence to somatic disorders in large remain to be identified [19,20].

Dysfunctional parenting and childhood exposure to interpersonal violence are among the most consistently documented risk factors for psychopathology worldwide [21,22]. Such exposure threatens or violates physical integrity, self-worth, confidence in personal capabilities, and trust in others. Common reactions include psychological distress, such as anxiety and depression, as well as social detachment and withdrawal [23]. We suspect that the social detachment which may follow exposure to interpersonal violence may be experienced as loneliness. An individual’s sense of loneliness is known to develop in the interplay between genetic predispositions and environmental influences, such as lack of parental attachment, peer rejection or bullying [24,25]. To our knowledge, the relationship between the range of interpersonal violence experienced by children and adolescents and loneliness remain to be explored. Loneliness, or perceived social isolation, has repeatedly been associated with an increased risk of adverse somatic health outcomes, independent of depression and anxiety, in adults [26-30]. In children and adolescents loneliness has been associated with headache [31], and identified as a common trigger of pain [1].

Social support, on the other hand, is a widely recognised protective factor against adverse mental and somatic health outcomes [19]. Early experiences of oneself as an individual who belongs, is loved, protected, and meaningfully supported in the face of need seem to lay ground for our perception of cohesion [32]. Whereas high levels of family cohesion foster resilience in children and adolescents, low levels have been linked to loneliness [33], posttraumatic distress [34], and headache [4]. Thus, family cohesion may constitute a general protective factor, potentially buffering the effects of exposure to interpersonal violence on health outcomes.

Another potential buffering factor is male sex. Adolescent girls have previously reported higher levels of loneliness and psychological distress and more headache complaints than their male peers [35,36]. The observed sex-biased discrepancy in prevalence rates could reflect differences in physiology, sociocultural role expectations or differential pathogenicity related to types of interpersonal violence, such as girls’ greater exposure to sexual abuse [37-40].

**Figure 1:**
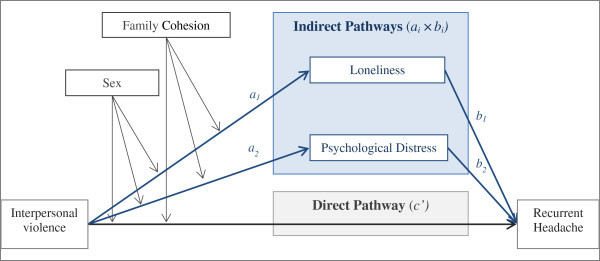
**Direct and indirect pathways linking exposure to interpersonal violence to recurrent headache, by sex and level of family cohesion **^**abc**^**.** Abbreviations: *a*_*i*_ × *b*_*i*_ (i = 1,2) = “indirect effect” of exposure to interpersonal violence on recurrent headache through loneliness (*a*_*1*_*x b*_*1*_*)* and psychological distress (*a*_*2*_*x b*_*2*_*); c’,* “direct effect” of interpersonal violence on risk of recurrent headache, adjusted for loneliness and psychological distress; CI, bootstrap 95% percentile confidence intervals presented, 10000 replications. Sex and family cohesion were modelled as moderators.

Taken together, we hypothesized that loneliness and psychological distress may pose as parallel mediators linking exposure to interpersonal violence to recurrent headache, whereas family cohesion and sex may serve as moderators buffering pathways. The current study was guided by this hypothetical model (Figure 1) [16]. The model was assessed in a large population-based cohort of adolescents.

## Methods

From 2006 until 2008, 10464 adolescents were invited to participate in Young-HUNT 3 (http://www.ntnu.edu/hunt), a population-based, cross-sectional cohort study of Norwegian youth in Nord-Trøndelag County. The study comprises a general health questionnaire, a clinical assessment, and a headache interview. The Norwegian Regional Committee for Medical and Health Research Ethics approved the study. Inclusion was based on informed written consent from participants aged 16 years and older, and from parents for those less than 16 years, in accordance with Norwegian law.

### Participants

In 2006, there were 128694 inhabitants in Nord-Trøndelag. Over 95% of these inhabitants were ethnic Norwegians, the work force was generally well-educated and unemployment was less than 3%. All 10464 adolescents who inhabited the county were invited to participate in the school-based study, including 5614 students in junior high, 4357 in senior high and 493 adolescents who did not attend school. Non-participation was mainly due to lack of enrolment, absenteeism, or participation in class activities outside of school [41]. Overall, 73% of the adolescents (7620/10464) responded to the headache interview and the questionnaire, 68% (7154) reported on family cohesion and 63% (6553) had no missing values for any of the variables included in the analysis. Most of the adolescents were from 13 to 18 years old, although age ranged from 12 through 20 years. Mean age of respondents in our sample was 16 years, two thirds lived with both parents, and the majority (>90%) of adolescents reported an average or above average family affordance [12].

During a school-hour, students completed a self-administered questionnaire that contained over 100 health- and lifestyle-related questions, including items on exposure to potentially traumatic events, loneliness, psychological distress, and family cohesion, in addition to background information on family structure and family economy (http://www.ntnu.edu/hunt/data/que). To assess adolescents’ recurring headache complaints according to type and frequency, a validated semi-structured clinical interview was conducted in association with a clinical examination within 1 month following completion of the questionnaire [42].

### Recurrent headache

All adolescents were asked if they had experienced recurring headache not caused by a cold (infection) or illness within the past 12 months. ‘Yes’ responders were read two descriptive texts of prototypic complaints for tension-type headache (TTH) and migraine, in accordance with the International Classification of Headache Disorders [43]. These participants were asked if they recognised either, both or neither descriptions as resembling their own complaints and further asked to specify the frequency of their head pain. Adolescents who reported ‘no recurrent headache’ or ‘complaints less than monthly’ were defined as having ‘no recurrent headache’, whereas all other headache frequencies were referred to as ‘recurrent headache’. The frequency of recurrent headache was categorised as monthly (1–3 days/month) or weekly (≥1 day/week) [12].

### Sociodemography

Information on gender and age was obtained from the Norwegian National Population Registry. The sociodemographic variable ‘family structure’ differentiated between ‘living with both parents’ versus ‘other’ [44]. Socioeconomic situation was labelled as ‘family economy’ and measured as the self-reported estimation of family affordance in comparison with most others, categorised as ‘above average’, ‘average’, and ‘below average’ [45].

### Interpersonal violence

In the present paper interpersonal violence was defined as *social actions subjecting an individual to intentional threats, use of physical force or power, that may cause immediate or long-term adverse developmental or health outcomes,* in line with the World Health Organization’s definition of the phenomenon [46]. Interpersonal violence encompasses both direct and indirect (witnessing) exposure [39]. Lifetime exposure to interpersonal violence was measured as i) Been subjected to violence (beaten or injured), ii) Seen others being subjected to violence, iii) Been subjected to unpleasant/disagreeable sexual acts by someone approximately your own age, iv) Been subjected to unpleasant/disagreeable sexual acts by an adult, and v) Been threatened or physically harassed by fellow students at school over a period of time, and labelled as exposure to *violence*, *witness to violence*, *sexual abuse by peer*, *sexual abuse by adult* and *bullying*, respectively. These measures were derived from the brief Young-HUNT3 lifetime trauma screen which is based on The University of California at Los Angeles Post-traumatic Stress Disorder Reaction Index (UCLA PTSD Reaction Index) [47], and accustomed to the Norwegian context. The UCLA PTSD Reaction Index was developed from the Survey of Children’s Exposure to Community Violence [48] and the Community Violence Exposure Survey (CVES) [49]. A sum-score of exposure to number of types of interpersonal violence was calculated to account for potential cumulative effects of exposure [50-52].

### Psychosocial factors

*Loneliness* was measured using a one-item variable termed ‘Does it happen that you feel lonely?’, in coherence with one-item measures used in prior studies of the phenomenon [31,36,53,54]. Answers were distributed across a five-point Likert scale, ranging from 1–5, where ‘*never or very rarely’* was coded as 1 and ‘*very often’* was coded as 5. In line with current recommendations [25], this measure focused solely on the emotional experience of loneliness, without assessment of hypothesised causes. *Psychological distress* was measured using a validated five-item, short-version instrument, the SCL-5. This measure was modified from the 25-item Hopkins’s Symptom Checklist (HSCL) subscale on anxiety and depression, which utilises a four-point Likert scale (ranging from 1 = *‘not bothered’* to 4 = *‘very bothered’*) [55,56]. The items measured whether adolescents had been bothered with feelings of i) fear or anxiety, ii) tension, distress or restlessness, iii) hopelessness about the future, iv) dejection or sadness, and/or v) excessive worry during the past 14 days. Cronbach’s α was 0.83 (girls) and 0.79 (boys). *Family cohesion* was measured using four items that were derived from the validated six-item family cohesion subscale from the Resilience Scale for Adolescents (READ) [57-59]. The four items included in the HUNT questionnaire were selected on recommendation from the developers of the original scale. Adolescents were asked to rate, on a five-point Likert scale, their agreement with four statements regarding i) shared family values, ii) personal well-being within the family, iii) shared positive expectations and hope in spite of adversity, and iv) support of each other over the past month. The mean score ranged from 1–5, where the highest measurable level of family cohesion was coded as 5. Cronbach’s α was 0.87 (girls) and 0.84 (boys).

### Statistics

The descriptive data and univariate analysis are presented by sex, according to frequency of recurrent headache (Table 1). Differences in prevalence-rates and mean values between the sexes were investigated in Pearson Chi square analyses. The interrelations between exposure to interpersonal violence, age, and psychosocial factors were explored through Pearson correlations (Additional file 1).

**Table 1:** **Recurrent headache by frequency in relation to exposure to interpersonal violence, loneliness, psychological distress and family cohesion, by sex, in 7154 adolescents **^**abcde**^

		**Recurrent headache**				
		**All**	**No**	**Monthly**	**Weekly**	
**Variables**	**N**	**n (%)/ mean (SD)**	**n (%)/ mean (SD)**	**n (%)/ mean (SD)**	**n (%)/ mean (SD)**	**p**
**Girls**	3639	1063 (29)	2576 (71)	614 (17)	449 (12)	
*Interpersonal violence*						
Sum-score	3543	0.4 (0.8)	0.3 (0.7)	0.5 (0.9)	0.7 (1.1)	<0.001^e^
Type						
Witness to violence	3578	627 (18)	383 (15)	125 (21)	119 (27)	<0.001^d^
Violence	3577	257 (7)	139 (6)	53 (9)	65 (15)	<0.001^d^
Bullying	3567	263 (7)	129 (5)	58 (10)	76 (17)	<0.001^d^
Sexual abuse by, peer	3578	200 (6)	109 (4)	52 (9)	39 (9)	<0.001^d^
Sexual abuse by, adult	3576	127 (4)	66 (3)	28 (5)	33 (7)	<0.001^d^
*Psychosocial factors*						
Loneliness	3523	2.3 (1.1)	2.2 (1.0)	2.5 (1.0)	2.9 (1.2)	<0.001^e^
Psychological distress	3606	1.6 (0.6)	1.6 (0.5)	1.8 (0.6)	2.0 (0.7)	<0.001^e^
Family cohesion	3639	4.2 (0.9)	4.2 (0.8)	4.1 (0.9)	3.9 (1.0)	<0.001^e^
**Boys**	3515	540 (15)	2975 (85)	386 (11)	154 (4)	
*Interpersonal violence*						
Sum-score	3396	0.5 (0.9)	0.5 (0.9)	0.6 (1.0)	0.8 (1.0)	<0.001^e^
Type						
Witness to violence	3422	949 (28)	781 (27)	115 (31)	53 (36)	0.022^d^
Violence	3424	427 (12)	339 (12)	57 (15)	31 (21)	0.001^d^
Bullying	3422	287 (8)	216 (7)	44 (12)	27 (18)	<0.001^d^
Sexual abuse by, peer	3429	79 (2)	62 (2)	13 (3)	4 (4)	0.255^d^
Sexual abuse by, adult	3428	54 (2)	43 (1)	9 (2)	2 (1)	0.395^d^
*Psychosocial factors*						
Loneliness	3328	1.9 (1.0)	1.9 (1.0)	2.2 (1.1)	2.4 (1.2)	<0.001^e^
Psychological distress	3456	1.3 (0.4)	1.3 (0.4)	1.5 (0.5)	1.6 (0.6)	<0.001^e^
Family cohesion	3515	4.3 (0.8)	4.3 (0.7)	4.2 (0.8)	3.8 (1.0)	<0.001^e^

^a^Recurrent headache is defined as headache monthly or more frequently; recurrence 1–3 times per month is defined as monthly headache, and weekly or more frequent recurrence is termed weekly.

^b^Variable range: Age, 12–20 y; Interpersonal violence sum-score 0–5; Loneliness, 1–5; Psychological distress, 1–4; Family cohesion, 1–5.

^c^Because of rounding, percentages may not total 100.

^d^Pearson Chi square test.

^e^ANOVA, analysis of variance.

The hypothetical moderated-mediation model of two parallel mediating (indirect) pathways that link exposure to interpersonal violence to recurrent headache through loneliness and psychological distress, moderated by sex and family cohesion, was presented in Figure 1. The model was explored using Conditional Process analysis, http://www.afhayes.com/, [60,61], which allows for assessment of multiple mediators working in parallel using recommended bootstrap methodology [62]. The estimated effect of exposure to interpersonal violence on recurrent headache through each of the two indirect pathways was calculated based on *a1 x b1* and *a2 x b2*. The regression coefficients *a*_1_ and *a*_2_ were estimated by separate linear regressions for each mediator (loneliness and psychological distress respectively) by exposure to interpersonal violence, adjusted for background variables and conditionally dependent on sex and family cohesion. *b*_*1*_*and b*_*2*_ were estimated as log odds ratios for recurrent headache by each of the mediators in a common logistic regression analysis, adjusted for background variables. Exp(*a1 x b1*) and exp(*a2 x b2*)) are interpreted and presented as odds ratios (ORs). The remaining direct effect (ORs) of PTIE exposure on recurrent headache (exp(*c’*)) was obtained from the logistic regression described above.

**Figure 2:**
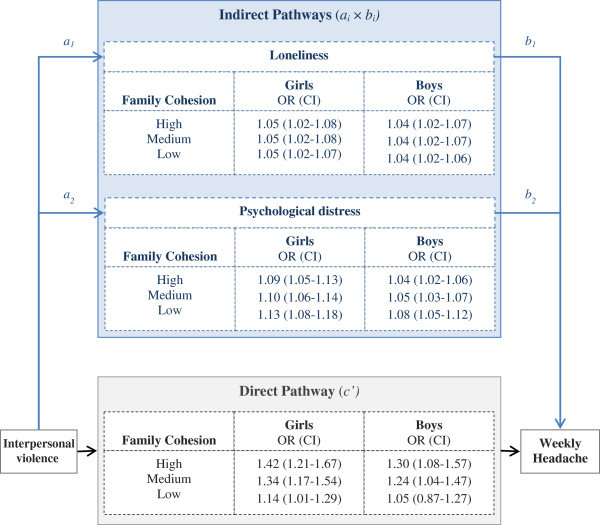
**Estimated direct and indirect pathways linking exposure to interpersonal violence to weekly headache, by sex and level of family cohesion **^**abc**^**. **^a^Study definitions and measures were defined in footnotes to Figure 1 and Table 1. ^b^Analyses were restricted to adolescents without missing values, 2820 (50) girls and 2823 (50) boys. The 910 cases that reported monthly recurrent headache were excluded. ^c^ Analysis were adjusted for family structure, family economy and age, with sex and family cohesion as moderators.

Conditional Process analysis, as described above, was performed in a complete case sample of 6533 (62%) of the 10464 adolescents who were invited to participate in the study. Weekly (Figure 2) and monthly (Additional file 2) headache served as separate outcomes. Adjusted ORs and 95% confidence intervals (CIs) are presented separately for girls and boys and for three levels of family cohesion corresponding to point-approximations of the 90^th^, 50^th^, and 10^th^ percentile, given by the values 5.00, 4.50, and 3.00, and labelled high, medium and low family cohesion, respectively. Thus, three effect-estimates for each of the sexes were calculated and presented for any given association. CIs were computed as bootstrap 95% percentile intervals based on 10000 replicated samples. Bootstrapping is a general procedure for computing CIs without making distributional assumptions [62]. CIs that do not include 1 indicate a significant indirect (mediating) or direct relationship. Consistent discrepancies of ORs with CIs between sexes, for a given indirect or direct association, indicate moderation by sex. Similarly, discrepancies of effect-estimates (ORs and CIs) between high, medium or low family cohesion indicate moderation.

Analyses were conducted using SPSS version 20, in combination with PROCESS [60].

## Results

In this population-based sample of adolescents exposure to interpersonal violence was common. Witnessing violence was the most frequently reported type victimization for both sexes (Table 1). Boys reported a higher exposure to violence (p <0.001), and girls reported more sexual abuse (p <0.001). Sexual abuse occurred rarely in boys. We found no significant difference in prevalence rates of bullying between the sexes (p = 0.116). Girls experienced significantly higher levels of loneliness and psychological distress and lower levels of family cohesion, compared to boys (all three p-values <0.001). As reported elsewhere, twice as many girls (29%) as boys (15%) reported recurrent headache and three times as many girls (12%) as boys (4%) reported weekly complaints [12]. Exposure to interpersonal violence was significantly associated with recurrent headache, with the highest degree of exposure reported by girls and boys who experienced weekly headache. This pattern was evident for prevalence of witnessing violence, violence exposure, and bullying in both sexes. In girls suffering from recurrent headache the observed prevalence of reported sexual abuse was doubled compared to peers without recurrent headache. Youth with recurrent headache reported more loneliness, more psychological distress, and lower levels of family cohesion in comparison to their headache-free peers. Loneliness, psychological distress and low family cohesion were most frequent in adolescents experiencing weekly headache.

All psychosocial variables were moderately correlated (Additional file 1).

To estimate the two hypothesized parallel indirect pathways between exposure to interpersonal violence and weekly headache through loneliness and psychological distress, conditionally dependent on sex and family cohesion (Figure 1), we conducted Conditional Process Analyses [60]. Figure 2 shows that the indirect pathways between interpersonal violence and weekly headache through loneliness (*a*_*1*_ × *b*_*1*_) were consistently and significantly above 1 for both sexes and across all levels of family cohesion. This finding indicated significant mediation through loneliness. The magnitude of the ORs for this indirect pathway through loneliness, corresponding to high, medium and low level of family cohesion for girls and boys respectively, differed only marginally. This observed overlap in ORs and corresponding CIs implied that there was no substantial moderation by sex or level of family cohesion of the indirect pathway between exposure to interpersonal violence and weekly headache through loneliness. Further, the pathway via psychological distress was explored. The analysis indicated mediation via psychological distress (*a*_*2*_ × *b*_*2*_). We observed an overlap of ORs and CIs estimating strength of associations according to level of family cohesion or sex, indicating no substantial moderation. Moreover, these analyses showed that despite the adjustment for the two explored, indirect pathways, the direct pathway (*c’*) between interpersonal violence and weekly headache remained consistently significant for girls at all levels of family cohesion, and for boys at high and medium levels of family cohesion.

The Conditional Process Analysis of pathways between interpersonal violence and monthly headache revealed a similar pattern to that described for weekly headache, although the associations were weaker (Additional file 2).

## Discussion

To our knowledge, the current study is the first to assess loneliness and psychological distress as parallel mediators that link exposure to interpersonal violence to an adverse somatic outcome.

The study findings are in support of the hypothesis that loneliness and psychological distress may pose as parallel mediating mechanisms that indirectly link exposure to interpersonal violence to recurrent headache in a general population of adolescents. We found no substantial moderating effect of family cohesion or sex.

The strengths of the current study are the large sample size, the overall high participation rate, the use of validated headache outcomes and the assessment of the impact of a range of commonly experienced types of interpersonal violence, loneliness, psychological distress, and family cohesion within a population-based cohort of adolescents.

The main limitation to this study is the retrospective, cross-sectional design, which hinders the assessment of temporality. The one-item measure of loneliness is a limitation. Measures of interpersonal violence exposure lacked event-specific information on severity and frequency of exposure. Generally, lack of intelligibility of questions increases confusion and variability in interpretation in respondents, typically leading to errors of omission (underreporting) [63-65]. However, although accuracy of memory processes and contemplations of disclosure may vary in relation to exposure to interpersonal violence, or somatic health status, adolescents exposed to interpersonal violence seem to be as accurate in their responses and testimonies as their non-exposed peers [63,66-74]. Errors of commission (over reporting) on the other hand seem to be infrequent in adolescents [63,65,66]. More comprehensive measures of exposure to interpersonal violence, loneliness, psychological distress, and family cohesion could have helped disentangle particularly potent risk or protective aspects of these broad features [75]. Although the prevalence rates of exposure to interpersonal violence and headache found in the present study were in the lower range of those observed elsewhere [35,76,77], and despite the accounted for restrictions related to design and measurements, it is likely that the main findings can be generalised to other adolescent populations.

Importantly, the current findings indicate that loneliness may represent an alternative (to psychological distress) mediating mechanism between exposure to interpersonal violence and recurrent headache, substantiating recent evidence from two cross-sectional studies of children and adolescents and adults. In the population-based child and adolescent study, the association between bullying and somatic complaints depended on concurrent experiences of loneliness [31]. Similarly, in the adult study, adverse experiences, parental maladjustment, and physical abuse in childhood were related to higher adult pulse pressure (a risk factor for adverse cardiovascular outcomes) only in participants who experienced a current high degree of loneliness [78].

Concerning the question of temporality, related longitudinal studies of adults have supported the directionality of associations presented in the hypothesised model (Figure 1). In healthy soldiers, exposure to interpersonal violence during war predicted loneliness [79]. Further, high baseline loneliness has been associated with a higher concurrent prevalence of pain and an increase in pain over time [80]. A recent study suggested that psychological reactions in individuals exposed to trauma initially predict somatic symptoms, including headache, but that a bidirectionality of associations evolve over time such that somatic symptoms predict persistence of adverse psychological reactions [81].

Current advances in neurophysiology support the notion that severe childhood adversity may overload the physiological stress response system and dysregulate cognitive and emotional processes in a cycle that fuels the chronification of headache [13,82]. Specifically, threats of our social selves may embed psychologically as experiences of shame or loneliness, related to neuro-endo-immunological dysregulation, and physical pain [29,30]. Thus, loneliness, defined as a painful experience of a lack of social belonging [27], may represent a key phenomenon bridging experiences of interpersonal violence to pain and headache. Further, loneliness and psychological distress could function as parallel internal reminders associated with onset, maintenance or exacerbation of pain, and vice versa. Additionally, the presented model may enhance our understanding of the psychosocial factors in play in relation to onset and maintenance of disorders modified through similar physiological mechanisms, such as other chronic pain conditions and fatigue [29,82].

## Conclusions

Psychological distress and loneliness play crucial roles in the relationship between exposure to interpersonal violence and recurrent headache. A biopsychosocial approach accommodating adolescents’ somatic, psychological and social needs could be beneficial in preventive public health efforts targeting recurrent headache. In clinical practice, it may be helpful to assess exposure to violence, sexual abuse, bullying, and psychosocial well-being in adolescents struggling with recurrent headache to tailor intervention and facilitate coping and recovery. Trauma-informed, social relationship-based interventions may represent a major opportunity to alter trajectories of recurrent headache.

## Abbreviations

CI: Confidence interval; ICHD-II: International classification of headache disorders, 2nd edition; OR: Odds ratio; TTH: Tension-type headache.

## Competing interests

Synne Øien Stensland, Siri Thoresen, Tore Wentzel-Larsen, John-Anker Zwart, and Grete Dyb declare no potential conflict of interest, real or perceived.

This work was supported by the Norwegian Centre for Violence and Traumatic Stress Studies and with a grant from The Norwegian Council for Mental Health, The Norwegian ExtraFoundation for Health and Rehabilitation, grant number 2009/2/0023. The Nord-Trøndelag Health Study (The HUNT Study), which is collaboration between HUNT Research Centre (Faculty of Medicine, Norwegian University of Science and Technology NTNU), Nord-Trøndelag County Council, Central Norway Health Authority, and the Norwegian Institute of Public Health planned, organized and financed the data collection. The funders were not involved in the study design, analysis, interpretation of data, the writing process, or the decision to submit the manuscript for publication.

## Authors’ contributions

SØS carried out the data processing, analyzed the data, drafted and revised the paper. GD and JAZ contributed to the integration of the headache interview and measures of victimization in the Young-HUNT3 Study. GD and ST wrote the original study protocol, applied for and received the grant for the study, and further participated in the conceptual and epidemiological modeling, analysis and writing of the manuscript. TWL contributed to the statistical analysis. JAZ participated in the design of the study and helped to write the manuscript. All authors read and approved the final manuscript.

## Supplementary Material

Additional file 1 Intercorrelation table of exposure to interpersonal violence, loneliness, psychological distress, family cohesion and age, by sex, in 7154 adolescents. Click here for file

Additional file 2 **Estimated direct and indirect pathways linking interpersonal violence to exposure to monthly headache, by sex and level of family cohesion **^**abc**^**.** Click here for file
