# Supplementary material for: Recurrent headache and interpersonal violence in adolescence: the roles of psychological distress, loneliness and family cohesion: the HUNT study
Source: J Headache Pain. 2014 Jun 10;15(1):35. doi: 10.1186/1129-2377-15-35 (PMC4085726; doi:10.1186/1129-2377-15-35)
Supplement: Additional file 1 — Intercorrelation table of exposure to interpersonal violence, loneliness, psychological distress, family cohesion and age, by sex, in 7154 adolescents. [file 1129-2377-15-35-S1.doc]

**Appendix 1**. Intercorrelation table of exposure to interpersonal violence, loneliness, psychological distress, family cohesion and age, by sex, in 7154 adolescents

| **Variable** | 1 | 2 | 3 | 4 | 5 |  | N | range | Mean (SD) |
| --- | --- | --- | --- | --- | --- | --- | --- | --- | --- |
| **Girls** | | | | | | | | | |
| Interpersonal violence | 1 |  |  |  |  |  | 3543 | 0-5 | 0.4 (±0.8) |
| Loneliness | 0.27*** | 1 |  |  |  |  | 3523 | 1-5 | 2.3 (±1.1) |
| Psychological distress | 0.35*** | 0.59*** | 1 |  |  |  | 3606 | 1-4 | 1.6 (±0.6) |
| Family cohesion | -0.24*** | -0.41*** | -0.42*** | 1 |  |  | 3639 | 1-5 | 4.2 (±0.9) |
| Age | 0.18*** | 0.10*** | 0.18*** | -0.05** | 1 |  | 3639 | 12-20 | 15.9 (±1.7) |
| **Boys** | | | | | | | | | |
| Interpersonal violence | 1 |  |  |  |  |  | 3396 | 0-5 | 0.5 (±0.9) |
| Loneliness | 0.23*** | 1 |  |  |  |  | 3328 | 1-5 | 1.9 (±1.0) |
| Psychological distress | 0.29*** | 0.52*** | 1 |  |  |  | 3456 | 1-4 | 1.3 (±0.4) |
| Family cohesion | -0.16*** | -0.31*** | -0.32*** | 1 |  |  | 3515 | 1-5 | 4.3 (±0.8) |
| Age | 0.19*** | 0.06** | 0.16*** | -0.05** | 1 |  | 3515 | 12-20 | 15.8 (±1.7) |

Pearson correlations (2-tailed). Due to missing data, sample sizes varied.

**p<0.01.

***p<0.001

**Appendix 2.** Estimated direct and indirect pathways linking PTIE exposure to monthly headache, by sex and level of family cohesion.^abc^

**Direct Pathway** (*c’)*

**Indirect Pathways** (*a_i_* × *b_i_)*

**Psychological distress**

*a_1_*

*b_1_*

**Loneliness**

*a_2_*

*b_2_*

1.03 (1.01-1.05)

1.03 (1.01-1.05)

1.03 (1.01-1.05)

1.03 (1.01-1.05)

1.03 (1.01-1.05)

1.03 (1.01-1.05)

High

Medium

Low

**Family Cohesion**

**Girls**

OR (CI)

**Boys**

OR (CI)

1.05 (1.03-1.08)

1.06 (1.03-1.09)

1.07 (1.04-1.11)

1.03 (1.01-1.04)

1.03 (1.02-1.05)

1.04 (1.02-1.07)

High

Medium

Low

**Family Cohesion**

**Girls**

OR (CI)

**Boys**

OR (CI)

1.20 (1.03-1.39)

1.16 (1.03-1.32)

1.07 (0.94 -1.22)

1.11 (0.96-1.28)

1.08 (0.95-1.23)

1.00 (0.86-1.16)

High

Medium

Low

**Family Cohesion**

**Girls**

OR (CI)

**Boys**

OR (CI)

**Monthly Headache**

**PTIE Exposure**

^a^Study definitions and measures were defined in footnotes to Figure 1.

^b^Analyses were restricted to adolescents without missing values, 2967 (49) girls and 2033 (51) boys. The 533cases that reported weekly or more frequent headache were excluded.

^c^ Analysis were adjusted for family structure, family economy and age, with sex and family cohesion as moderators.
