# Supplementary material for: Recurrent headache and interpersonal violence in adolescence: the roles of psychological distress, loneliness and family cohesion: the HUNT study
Source: J Headache Pain. 2014 Jun 10;15(1):35. doi: 10.1186/1129-2377-15-35 (PMC4085726; doi:10.1186/1129-2377-15-35)
Supplement: Additional file 2 — Estimated direct and indirect pathways linking interpersonal violence to exposure to monthly headache, by sex and level of family cohesion abc . [file 1129-2377-15-35-S2.doc]

**Appendix 2.** Estimated direct and indirect pathways linking exposure to interpersonal violence to monthly headache, by sex and level of family cohesion.^abc^

**Direct Pathway** (*c’)*

**Indirect Pathways** (*a_i_* × *b_i_)*

**Psychological distress**

*a_1_*

*b_1_*

**Loneliness**

*a_2_*

*b_2_*

1.03 (1.01-1.05)

1.03 (1.01-1.05)

1.03 (1.01-1.05)

1.03 (1.01-1.05)

1.03 (1.01-1.05)

1.03 (1.01-1.05)

High

Medium

Low

**Family Cohesion**

**Girls**

OR (CI)

**Boys**

OR (CI)

1.05 (1.03-1.08)

1.06 (1.03-1.09)

1.07 (1.04-1.11)

1.03 (1.01-1.04)

1.03 (1.02-1.05)

1.04 (1.02-1.07)

High

Medium

Low

**Family Cohesion**

**Girls**

OR (CI)

**Boys**

OR (CI)

1.20 (1.03-1.39)

1.16 (1.03-1.32)

1.07 (0.94 -1.22)

1.11 (0.96-1.28)

1.08 (0.95-1.23)

1.00 (0.86-1.16)

High

Medium

Low

**Family Cohesion**

**Girls**

OR (CI)

**Boys**

OR (CI)

**Monthly Headache**

**Interpersonal violence**

^a^Study definitions and measures were defined in footnotes to Figure 1.

^b^Analyses were restricted to adolescents without missing values, 2967 (49) girls and 2033 (51) boys. The 533cases that reported weekly or more frequent headache were excluded.

^c^ Analysis were adjusted for family structure, family economy and age, with sex and family cohesion as moderators.
